# Degradomics for large-scale mechanistic insights on proteases and proteolysis in human health

**DOI:** 10.1111/febs.70504

**Published:** 2026-03-23

**Authors:** Daniel R. Martin, Sumit Bhutada, Suneel S. Apte

**Affiliations:** Department of Biomedical Engineering, Cleveland Clinic Research, Cleveland Clinic, Cleveland, OH, USA

**Keywords:** mass spectrometry, protease, proteolysis, proteomics, terminomics

## Abstract

Tissue breakdown, especially extracellular matrix or secretome breakdown is a significant aspect of physiological remodeling and disease processes in solid organs, with profound structural and regulatory impact. Tissue and circulating proteins undergo breakdown through a chemical process mediated by proteases, that is, hydrolysis of peptide bonds. Proteolysis has immense biological impact because it is irreversible, results in protein inactivation or activation, and can generate fragments with new functions to expand the functional genome. The traditional focus on a few proteases of interest against candidate substrates provided limited insights into the proteolytic landscape of human diseases. In contrast, innovations in protein terminomics workflows, tandem mass spectrometry and data handling now routinely permit identification of proteolytic events and proteases on the proteome scale, the degradome. The unbiased, *de novo* elucidation of disease degradomes, termed forward degradomics, has increased the number of known *in vivo* proteolytic events, despite only limited application to human disease to date. In reverse degradomics, activities of proteases are elucidated individually, but also at the proteome scale by digesting protein libraries sourced from tissues and cell secretomes, or by comparing the degradomes of protease-deficient/overexpressing and parental cells. Integration of forward and reverse degradomes precisely defines protease primary mechanisms in disease. Cross-disease degradome analysis can define disease-relevant, protease-specific biomarkers, identify proteases as appropriate therapeutic targets, and predict cross-organ impact of protease inhibitors. A systematic effort to map disease degradomes, that is, a prospective human degradome project would generate a comprehensive proteolysis knowledgebase for diagnostics and therapeutics.

## Introduction

Decoding protein post-translational modifications, especially of extracellular matrix (ECM) components, remains a pressing challenge for the postgenome era. One such modification is proteolysis, which being irreversible, places an indelible mark on ECM by generating proteoforms with new (neo) N- and C-termini. Proteolysis can activate gene products, inactivate them, release bound ligands, modify adhesive sites, and generate bioactive fragments. It is widespread and underestimated; one study found that nearly 50% of proteins in skin had stable cleavage products [1], reflecting the ongoing remodeling of both healthy and disease tissues. ECM proteolysis has enormous biological significance in disease, not only in those conditions where tissue destruction is a principal feature, for example, osteoarthritis, osteoporosis, invasive and metastatic cancers, pulmonary emphysema, and aortic aneurysms, but also in other common disorders, including autoimmune/inflammatory diseases, myocardial infarction, atherosclerosis, and fibrotic conditions affecting a variety of organs. Since cells and their ECM comprise a functional unit, with ECM acting as a repository of information, proteolysis can potentially edit and/or release this information, with powerful regulatory consequences. Despite this impact, the role of proteases in ECM dynamics is poorly resolved in human disease, with few studies conducted at the proteome-wide level. Traditionally, protease research relied on digestion of selected substrates or synthetic peptide libraries by hand-picked proteases resulting in a limited view of disease-relevant cleavage events. Traditional hypothesis-driven research typically also focused on the trendiest proteases and substrates. For example, the decade between 1995 and 2005 saw a disproportionate focus on MT1-MMP in the context of cancer and the proteolytic activity termed aggrecanase (ADAMTS4 and ADAMTS5) in osteoarthritis. During this period and subsequently, much of the protease and ECM space received far less attention.

Association of proteases with diseases has often relied on mRNA detection by RT-qPCR, cell lineage-specific gene expression described by transcriptomics and protein localization by immunostaining. None of these are convincing surrogates for protease activity, since proteases are typically synthesized as zymogens which require activation and most have ambient inhibitors. In the context of the current emphasis on transcriptomics in biomedical research, the cellular origin of proteases may also be misleading, since secreted proteases act noncell-autonomously; an illustrative example is that of clotting factors, most of which are proteases synthesized in the liver that circulate in blood and are active at a distant injury site. The only true measure of ECM proteolysis is a cleaved peptide bond or protein fragmentation observed by gel electrophoresis, but the landscape of proteolytic cleavages is still rather barren, especially in human disease. Which sites in ECM proteins are cleaved? Which proteases are the effectors? How active are these proteases in healthy vs disease tissues? Are specific proteolytic cleavages unique to disease states and what can they tell us about disease status, prognosis and responsiveness to treatment? Since few ECM-modifying proteases have stringent consensus cleavage site sequences, uncovering protease activity has largely not been amenable to *in silico* prediction and requires experimental evidence.

**Fig. 1.**
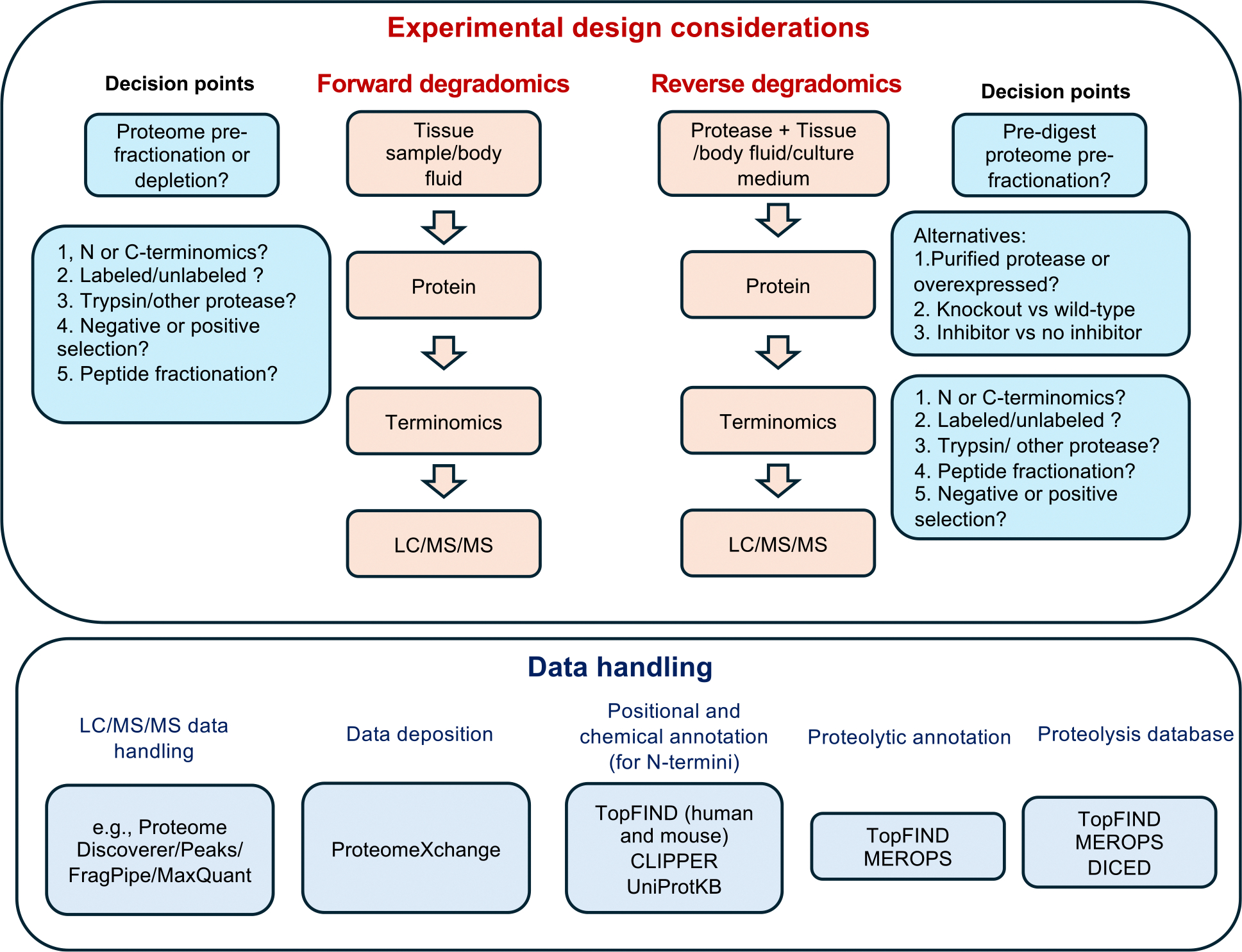
Overview of the experimental design considerations for forward and reverse degradomics with key decision points indicated and summary of the main data analysis aspects.

The advent of N-terminomics, especially the widespread application of Terminal Amine Isotopic Labeling of Substrates (TAILS) [2], demonstrated that despite inherent technologic limitations and ongoing challenges outlined later in this review, it was possible to develop a proteome-scale ‘degradome’ using tissue, cells, or body fluids as starting material and greatly expand our knowledge of proteolysis. This primary annotation of naturally occurring proteolysis in complex proteomes such as tissues and body fluids, using a variety of methods outlined in Table 1 is referred to as ‘forward degradomics’. ‘Reverse degradomics’ uses quantitative strategies applying the same methods for protease-selective, unbiased substrate discovery using mass spectrometry after digestion of a proteome containing relevant substrates with a protease of interest. Higher abundance of peptides with neo-termini compared to those generated in digests with an inactive form of the protease or with an added inhibitor identifies sites of cleavage by the protease. Reverse degradomics can also be undertaken by quantitative comparison of proteomes of cell cultures and tissue from gene knockouts or with engineered over-expression against corresponding wild-type samples (Fig. 1). The purpose of this review is to provide an overview of key methodological concepts and experimental design considerations and illustrate the application of degradomics directly to human health challenges using representative examples. Degradomics using experimental animal models of human disease is not covered in this review unless it serves to illustrate specific concepts.

## DEGRADOMICS strategies employing TERMINOMICS with protein labeling

Terminomics uses specialized proteomics approaches for defining protein termini on the proteome scale. The simple principle underlying terminomics is that every intact protein and proteoglycan has one N and C terminus. Each proteolytic cleavage generates an additional N- and C terminus. Degradomics is therefore based on N- and C-terminomics which identifies protein termini and based on the location of the observed terminally labeled peptides with respect to the protein translation start, determines whether they are proteolytic in origin. When signal peptide and propeptide processing generates termini, these, along with the start codon and last amino acid, are referred to as natural termini. Protein termini other than these, typically located further downstream in the protein sequence, are referred to as neo-termini and are presumably proteolytic, since unannotated alternative start sites are rare. Thus, positional peptide annotation is an important step in degradomics (Fig. 1). N-termini are more chemically reactive, and since, at least in theory, both N- and C-terminomics should identify the same cleaved peptide bonds, N-terminomics is more widely used. A number of N- and C-terminomics degradomics strategies are listed in Table 1.

Terminomics relies on liquid chromatography, tandem mass spectrometry (LC–MS/MS), which requires explanation of terms commonly used in this technology. The typical application of LC–MS/MS to proteins is as ‘*shotgun proteomics*’, where the proteome of interest is digested, typically with trypsin, to generate peptides, which are injected into the mass spectrometer, where mass/charge ratio and fragmentation pattern provide the peptide sequence [3]. Trypsin cleaves proteins predictably after lysine and arginine residues, providing predictable peptides between 7 and 50 amino acids. Methods that utilize peptides to identify proteins in this way are also referred to as ‘*bottom-up*’ proteomics [3,4]. Until recently, such analysis has commonly used *data-dependent acquisition (DDA)*, where a limited number of the most abundant ions detected in the MS1 phase (the data) are selected for a second round of fragmentation (MS2) [3,4]. While DDA is robust, low-abundance ions are frequently underrepresented, because the mass spectrometer favors the most abundant peptides. This challenge is particularly evident in analysis of complex mixtures of molecular components with a high dynamic range of abundance such as tissues and body fluids and is referred to as *high proteome complexity*. It can be mitigated by tissue extraction methods that favor isolation of specific component groups (prefractionation, for example, proteoglycan isolation by ion-exchange chromatography), by depletion of the most abundant proteins, or offline fractionation of peptides prior to LC–MS/MS (Fig. 1). Recently, use of *data-independent acquisition (DIA)* is expanding [5,6]. In DIA, the mass spectrometer sequences all peptides detected in overlapping narrow mass windows, regardless of their relative abundance. This is advantageous over DDA methods, but it presents a new challenge in distinguishing individual peptide sequence information from the spectra of tens to potentially hundreds of different peptides. This computational challenge has been addressed over the past decade with a focus on identifying peptides that specifically - arise from trypsin digestion, which is typically used in bottom-up proteomics [7]. Thus, proteolytic peptides, which can have only one or no tryptic termini, can still be missed because of their lower abundance and computational suboptimization. Therefore, approaches for annotating and enriching proteolytic termini/peptides were necessary and provide the basis of degradomics.

### Experimental design considerations

In Fig. 1, we provide an outline of experimental design considerations, which in our experience, heavily influence the outcome. In terminomics with labeling, whether used for forward or reverse degradomics, there are a number of decision points to be considered (Fig. 1). A critical step after protein precipitation in TAILS and HUNTER, for example, is experimental labeling of termini at the level of proteins, before they are reduced to peptides using trypsin, otherwise the existing termini would be invisible against the background of new tryptic termini. Labelling not only adds a mass tag, but also blocks the N-termini, whereas tryptic peptides retain reactivity. Proteolytic peptides are normally of lower abundance in the mixture of peptides generated by trypsin or other ‘working’ proteases; therefore, an enrichment step is usually needed, constituting a *de facto* fractionation step. The methods used for enrichment fall into two categories: positive or negative selection of the labeled/proteolytic peptides. In some conditions, neo-termini are identifiable in shotgun MS datasets by semi-tryptic data analysis, which we discuss separately as an unlabeled degradomics approach. Other review articles that focus on terminomics technologies [8,9] and the original articles cited in Table 1, provide greater detail of each workflow. Terminomics workflows for tissue biopsies, or of body fluids as ‘liquid biopsies’ have also been the subject of excellent reviews [10–12].

The time from tissue harvest to freezing should be minimal and broad-spectrum protease inhibitors should be added when tissue is thawed for forward degradomics. Prior to reverse degradomics, inhibitors for the protease class being tested should be omitted, but inclusion of inhibitors of other classes can be added to minimize ‘indirect’ cleavages. For reverse degradomics, library selection is a crucial factor in determining which substrates are discovered and how readily. A key concept in defining the repertoire of cleaved secreted proteins is that the cell source is less relevant than its secretome/matrisome, for example, any cell line with an overlapping matrisome repertoire could be used for determining activity of a protease expressed by vascular endothelium or smooth muscle. Because of protease redundancy, tissues or cells with knockout of only one gene of a homologous pair may have sufficient background proteolysis from the intact homolog to conceal authentic proteolytic events. In such instances, double knockouts should be used [1,13]. Tissues such as cartilage which has low cellularity, have a very high percentage of ECM and are well-suited for uncovered ECM proteolysis [14]. On the other hand, highly cellular tissues such as solid tumors have a large dynamic range arising from high cellular protein abundance. For such tissues, decellularization, extensive peptide fractionation, or other measures to compress the dynamic range, should be considered.

Terminomics traditionally required a minimum of 150–300 μg of total protein, two orders of magnitude greater than that required for shotgun proteomes, because only a fraction of the peptides are proteolytic in origin and 90% of the peptides, typically tryptic peptides, are discarded during the enrichment step. However, some workflows such as HUNTER (Table 1) and analysis on the newest mass spectrometers can provide high quality data with much less protein. Relevant to sample proteome complexity, body fluids typically contain several serum-derived proteins such as albumin at high concentration, leading some to employ depletion of the most abundant proteins in order to compress the dynamic range. Offline peptide fractionation by chromatographic methods and separation within the mass spectrometer using high-field asymmetric waveform ion mobility spectrometry (FAIMS) have also been shown to increase the yield of terminome peptides in complementary ways [15]. High dynamic range appears to be less of a hindrance to detection of low-abundance peptides on the newest mass spectrometers with improved speed and depth of analysis.

Proteome isolation for forward degradomics (on ice and with protease inhibitors) can be done with or without fractionating tissue proteins or enrichment of the matrisome, for example, detergent extraction of tissue to minimize cellular proteins, followed by salt extraction (for relatively soluble ECM proteins) and guanidine extraction of insoluble ECM pellets. In addition to serial extraction of ECM and associated molecules, selective enrichment of N-glycosylated proteins (with lectins) and proteoglycans (using anion exchange) could be considered to overcome the large dynamic range in tissues and body fluids. In methods such as TAILS and HUNTER, N-terminal labeling using formaldehyde (reductive dimethylation) provides up to three isotopes for labeling samples individually for duplexing [16]; for experiments that involve quantitative comparison of a larger number of samples, they can be multiplexed for LC–MS/MS analysis after labeling individually with isobaric TMT tags. In TAILS, tryptic peptides are removed by negative selection with hyperbranched polyglycerol aldehydes (HPG-ALD) and the residual N-terminome is used for LC–MS/MS. A portion of the pre-HPG-ALD sample (pre-TAILS) is used for full proteome mapping and usually yields additional peptides with neo-termini. Labeling and pull-down efficiency are easily determined from the TAILS and pre-TAILS samples for experimental optimization of troubleshooting.

Shedding of the ectodomains of transmembrane proteins is an important area of protease research because of its broad biologic impact and presents a special challenge for degradomics. Transmembrane topology must be considered in attempting to identify the proteolytic products, for example, type I transmembrane proteins have an extracellular N terminus and single ectodomain cleavages would be undetectable by N-terminomics of culture medium or body fluids, whereas the converse is true for type II proteins and the orientation of some multi-pass membrane proteins is not yet known. Methods have been developed to define N-termini displayed on the cell surface [17] or identify the shed proteins, although not their termini [18]. Tsumagari et al used both N- and C-terminal peptide enrichment to identify a number of shed proteins, including several whose shedding was responsive to a metalloprotease inhibitor [19]. Thus, an important consideration in the context of protein shedding experimental design is whether to analyze cell lysate or medium or both using terminomics.

### Degradomics data processing

Following data acquisition, mass spectra are deconvolved by dedicated proteomics software (see Table 2) to rigorously identify and quantify peptides. For DDA experiments, options include commercial platforms such as Proteome Discoverer^™^, which is native for Orbitrap instruments and Spectromine^™^, Peaks, and open-source tools like maxquant and Fragpipe [20]. For data-independent acquisition (DIA) analysis, spectronaut^®^ is widely used, as are available open-source alternatives including fragpipe [21] and DIA-NN [22]. The latest protein database appropriate for the species of interest is downloaded from UniProtKB/Swiss-Prot for up-to-date searches when the proteome species is known (see section on host–pathogen interactions). Since trypsin is widely used as the working protease, searches are usually set to semi-tryptic, which additionally identifies semi-tryptic C-termini. Carbamidomethylation on cysteines is usually specified as a fixed modification (given standard protein disulfide reduction and alkylation), with variable modifications set to methionine oxidation, N-terminal acetylation, and glutamate/glutamine to pyroglutamate conversion on the peptide termini and isobaric tags or dimethyl on peptide N-termini and lysine. Trypsin missed cleavages are typically set to at least 2, since the blocked lysines resulting in N-terminal labeling methods such as TAILS and HUNTER will result in longer peptides than is usual after trypsin digestion.

*De novo* sequencing is a specialized mass spectrometry data analysis in which peptide sequence is deduced without a reference database [23]. It is valuable in several contexts, such as expanding the yield of identified peptides when an organism’s proteome is unavailable or incompletely annotated, for identification of non-tryptic peptides, such as immunopeptides, or analysis of mixed proteomes, for example, infected tissues. New sequencing algorithms and deep learning packages for *de novo* sequencing are emerging [24,25] that are likely to lead to increased adoption in terminomics, particularly when used in species other than humans and mice.

### Annotating peptides on proteins to identify cleavage sites

Downstream tools available for peptide annotation (see Table 2) include MANTI [26] used for MaxQuant output, TermineR [27] for FRAGPIPE and CLIPPER [28] for Trans Proteomics Pipeline [29]. CLIPPER 2.0 [30] uses Proteome Discoverer and Spectronaut data and can be customized for any software that provides information for normalized peptide intensities, accession number, and modifications. These tools can be used for peptide annotation, statistical analysis, and limited data visualization. Once annotation is complete, Perseus [31] can also be used to perform statistics and data visualization. Both N/C-terminal labeling efficiency and the proteolytic peptide enrichment factor should be estimated whenever possible for method optimization. After initial data analysis in reverse degradomics identifies neo-termini, it is necessary to filter out those that arise in proteins that do not occupy the same cellular compartment as the protease of interest. Cytosolic and nuclear proteins, for example, would not typically be relevant substrates of a secreted protease, although intracellular ‘moonlighting’ by secreted matrix metalloproteinases has been previously identified [32].

### Public resources for terminomics

Uniprotkb is the major resource for protein and peptide annotation, specifying signal peptide, transit peptide, and propeptide sequences and protein isoforms arising from alternative translation start sites or splicing events, which can create distinct termini for a given protein. UNIPROTKB provides access to structural information regarding protein domains, post-translational modifications and functional regions which are useful as a reference map, lists known genetic variants and summarizes known functions. MEROPS and TopFIND 4.1 are curated databases that compile experimentally validated cleavage sites allowing forward degradome annotation with known cleavage sites. MEROPS [33] is a peptidase and inhibitor database that organizes proteases into families and clans based on sequence homology, identifying evolutionary relationships and functional similarities. It provides extensive descriptions for each protease, including known substrates, specific inhibitors, and relevant scientific literature. It is potentially useful to identify candidate proteases responsible for observed cleavage events based on substrate specificity patterns and evolutionary relationships. TopFIND 4.1 is a major knowledgebase for N-terminomics research containing protein N-termini from experimental observations, predicted cleavage sites, and alternative translation start sites and splicing events [34]. The database retrieves information from various resources such as experimental N-terminomics, UNIPROT, MEROPS, and community submissions, then categorizes termini as either ‘observed’ (from direct experimental evidence) or ‘inferred’ (from predicted protease cleavage sites or other computational analyses). TopFIND 4.1 provides additional tools such as PATHFINDER which can explore complex, indirect proteolytic pathways.

Database of Identified Cleavage Sites Endemic to Diseases (DICED) [35] is a recently introduced resource for terminomics that provides open access to N- and C-terminal peptide data from diseased tissues and reverse degradomes obtained by the authors. The database can be searched using UNIPROT accession number, protein name, gene symbol, or up to 100 peptide sequences to find experimentally identified cleavage sites. DICED is intended to facilitate identification of disease-associated protease activity patterns and comparisons of proteolytic events across diseases, tissues, body fluids, and species.

## Label-free TERMINOMICS and related approaches

Most LC–MS/MS-based shotgun proteomics analyses use trypsin as the working protease. Since trypsin specifically cleaves after Arg or Lys, peptides that lack predicted ‘tryptic’ termini (at either or both of the N or C terminus) are very likely to be proteolytic in origin. Bell and Overall found that 36% of the N-termini identified by tails could also be identified by semi-tryptic peptide searches [36]. Since many proteases cleave after Arg or Lys, semi-tryptic peptide analysis alone will miss such cleavages. Nevertheless, semi-tryptic searches were productively used for targeted analysis to identify fibronectin as a substrate of the proteases ADAMTS9 and ADAMTS16 [33,34]. Ryden et al. analyzed shotgun proteomes of synovial fluid samples taken from patients with knee osteoarthritis and identified numerous nontryptic and semi-tryptic peptides indicative of several known proteolytic activities [32]. They developed the proteasy R package to retrieve experimentally annotated proteases and respective cleavage site data from the merops database for facile cross-analysis with shotgun proteomes. Such terminomics data mining could be used more widely to take advantage of numerous archival shotgun proteomics datasets deposited in ProteomeXchange.

A quantitative LC–MS/MS-based semi-tryptic peptide identification approach, termed the *z*-score method [37], was developed to attribute proteolytic events to specific proteases without prior labeling and enrichment. This label-free approach uses background (tryptic) peptide ratios and abundance to determine significance levels of semi-tryptic peptides. As in other semi-tryptic approaches, trypsin-like cleavages are obscured, potentially providing a skewed view of protease specificity. Tryptic peptides with reduced abundance can support the presence of a cleavage site in that peptide. The experimental set-up requires parallel digestion of a substrate or small library of substrates with an active and inactive protease. The inactive protease is ideally genetically modified but can also be heat denatured or chemically inactivated. Ideally, a proteolytic event should produce 3 unique peptide sequences, that is, 2 peptides arising from the N- and C-terminal side of the cleavage with higher abundance in the active protease digest and a tryptic peptide that spans the cleavage site and has lower abundance in the active protease digest. Therefore, both tryptic and semi-tryptic peptides undergo statistical analysis to identify proteolytic events. A tryptic peptide with higher abundance in the inactive protease digest supports the occurrence of proteolytic degradation within its sequence but does not provide the precise cleavage site. To disclose trypsin-like activities, an alternative working protease such as Glu-C can be employed with appropriate semi-Glu-C data searches. Table 1 lists several terminomics approaches that do not utilize N- or C-terminal labeling but are more complex than semi-tryptic site analysis from shotgun proteome datasets since they employ one or more additional processing/enrichment steps.

### Peptidomics

Label-free detection and sequencing of small peptides can complement degradomics methods that use bottom-up proteomics since initial size fractionation in the peptidomics sample preparation workflow, such as by molecular weight cut-off filters, excludes large proteins and large fragments. Selecting a pool of small peptides in this way reduces sample dynamic range and increases the likelihood of peptide detection; however, peptide identification requires database searches with no protease specified and may require *de novo* sequencing. Furthermore, peptidomics is limited to small fragments of proteins and may rely on endopeptidase cleavages occurring sufficiently near each other. It may also be necessary to distinguish such products of regulated protein processing from protein degradation products. In a recent study, saliva from patients with oral squamous cell cancer was used for a coupled peptidome-proteome analysis [38], which identified numerous proteolytic peptides and demonstrated a reduction in protease inhibitors in saliva from patients with lymph node-positive cancer. Plasma and lymph peptidomics have been used to identify circulating endogenous or self-antigens relevant to the major histocompatibility complex and hence immune tolerance and autoimmune conditions; many of these may be proteolytic in origin [39]. Clement et al [40] found that in addition to the endosomal processing pathway that generated cathepsin-cleaved peptides, the peptidome eluted from HLA-DR1 receptor on dendritic cells contained numerous small peptides that were attributed to diverse metalloproteases such as MMPs and ADAMs. Peptidomics of a size-selected protein fraction (< 5 kDa) identified numerous peptides in human pancreatic cyst fluid, the majority arising by cleavage of plasma proteins [41]. Since pancreatic cysts contain several proteases, the peptides report their activity, which is potentially useful for diagnostic purposes. Alignment of semi-tryptic peptides and peptidomes to the human plasma peptide atlas, for example, would be useful for prioritization of cleaved peptides likely to be detected in the circulation [42].

### Protein N-terminal sequencing

Despite advances in mass spectrometry techniques, N-terminal protein sequencing using Edman degradation [36] has a necessary role in investigating ECM proteolysis. This is because LC–MS/MS detection of peptides with extensive PTMs or with Arg residues adjacent to or distant from the cleaved site (resulting in peptides outside the set instrument mass range) may be challenging. N-terminal protein sequencing was used to uncover the canonical cleavage site of adamts proteases in the interglobular region of aggrecan, and to identify additional cleavages in the heavily modified central regions of aggrecan and versican [43,44]. Although these sites were mapped before the advent of mass spectrometry terminomics, their discovery would be unlikely using mass spectrometry-based terminomics as discussed later in this review. Protein fragments used for Edman degradation must be pure and in sufficient quantity and typically need to be bound to polyvinylidene fluoride membranes after electrophoretic separation. The N terminus is determined by stepwise amino acid labeling and cleavage, followed by chromatography of the eliminated residues to determine their molecular mass.

## Investigation of human health and diseases using DEGRADOMICS

### Pathogen–host interactions

Bacterial and helminth (parasitic worm) pathogens produce proteases that can act as virulence factors by modulating host immune responses and destroying host tissues. Peptides generated by pathogen proteases are likely detected during degradomics of infected tissues or healthy surface tissues and their microbiomes, but the responsible proteases can be difficult to identify, in the absence of reverse degradomics analysis of pathogen proteases. This is especially true in mixed pathogen colonization, such as of open wounds or in conditions which have similar clinical presentations despite infections with different pathogens, such as infective endocarditis. Infective endocarditis is a dangerous condition that results from systemic infection with a variety of pathogens and is characterized by formation of wart-like vegetations on the surface of heart valves, which are locally destructive to valve and surrounding tissue, interfere with valve function and may embolize to cause infective thrombi at distant locations. Among responsible pathogens, the most common is *Staphylococcus aureus*, although Gram-negative organisms and fungi are also implicated. The vegetations represent the outcome of a host–pathogen interaction on the endothelial surface of heart valves. Martin et al determined the degradome and proteome of vegetations and found a large number of proteolytic peptides [45], but attribution to proteases was not undertaken. Reverse degradomics of the staphylococcal V8 protease (commonly used as endoproteinase GluC for proteome digestion in proteomics workflows) against the neutrophil proteome was achieved by Mustor et al [46], using TAGS-CR terminomics (Table 1), identifying 350 new substrates, including proteins with a critical role in neutrophil defense. Using aminobiotinylation and TAILS against the plasma proteome, 90 substrates directly relevant to *S. aureus* pathogenesis were identified including proteins of the clotting cascade, protease inhibitors and the complement system [47], consistent with proteome analysis of vegetations, which showed an abundance of cleaved circulating and neutrophil-derived proteins [45].

Microbial proteases may be involved in homeostatic proteolysis occurring at interfaces having a substantial microbiome population such as colonic, bronchial, and vaginal mucosa [48]. Thus, proteolytic activity from healthy microbiomes could very well contribute to good health and this could be an area of future research. For example, meprin β-mediated MUC2 cleavage regulates mucus detachment in the intestine and prevents bacterial overgrowth [49]. *Porphyromonas gingivalis* infection of neutrophils or processing of the neutrophil proteome by its protease RgpB, a gingipain, led to cleavage of several azurophilic granule proteins whose neoepitopes, translocated to the surface, activated macrophage internalization of live neutrophils. Macrophages with entrapped neutrophils had subdued proresolution pathways, hindering rapid resolution of inflammation [50].

Degradomics was used to elucidate the role of the 3CL main protease (3 CL M^pro^) produced by the SARS-CoV-2 virus responsible for the recent COVID-19 pandemic. Pablos et al. used tails for degradomics to identify cleaved human proteins in human embryonic kidney cells and IFN-α treated human lung epithelial (BEAS-2B) cells infected with SARS-CoV-2 to identify 102 cleavages in 101 proteins [51]. This analysis identified an impact on the Hippo signaling pathway, thereby affecting cell morphology, mechano-transduction, tissue growth, and regeneration. One of the manifestations of COVID-19 is a hypercoagulable state. Pagotto et al. showed that M^pro^ could directly activate coagulation factors VII and XII, each a protease [52]. Degradomics also showed that 3 CL M^pro^ had an extended secondary specificity for cleavage at R↓X that was distinct from its known preference for LQ↓X sites [51,52].

Although not a host–pathogen interaction, snakebites by Viperidae species, which inject hemorrhagic venoms, are an important public health challenge and significant cause of mortality in many parts of the world. The envenomation exposes the human proteome to proteolysis at the bite site and in distant organs such as the kidney [53]. Hemorrhagic snake venoms contain an array of proteases, some of which are metalloproteinases collectively grouped as reprolysins, whose catalytic domains resemble those of human ADAMs and ADAMTS proteases. Administration of venom from the highly venomous south American pit viper *Bothrops jararaca* identified thousands of proteolytic cleavages in the kidneys, with a large number attributed to activation of host proteases [53]. An evolving profile of proteolytically cleaved sequences with time after the envenomation suggested activation of a number of host proteases or destruction of their inhibitors. A high frequency of cleavages observed after asparagine residues specifically suggested activation of endogenous legumain [53]. In another study, the activity of the protease HF3 from this snake species was defined against human plasma after depletion of abundant components and peptidomics was applied to identify the proteolytic products [54]. Such analyses are valuable because they could provide biomarkers for severity of envenomation and could assist in development and testing of antivenoms, including *de novo* designed proteins [55].

### Human tissue and fluid degradomes

Animal models of human diseases have been subjected to terminomics, such as a rat ischemia–reperfusion injury model [56], a genetic pancreatic tumor model [57] and an inflammatory colitis model [58], but because such models generally do not faithfully replicate human pathogenesis, this section will focus on direct analysis of human material. Surprisingly, little terminomics analysis has been done in human tumors. Song et al. developed a cleavable 2-pyridinecarboxyaldehyde probe (2PCA-Probe) for N-terminome determination and a new marker identifying N-terminal chemical labeling with the 2PCA-Probe and applied the method to identify 4686 N-terminal peptides in colorectal cancer and adjacent tissues [59].

Lorentzen et al. utilized a label-free N-terminomics approach after digestion of proteins from carotid artery plaques obtained from 21 patients via endarterectomy [60]. The study identified over 4000 N-terminal peptides determined to arise from proteolysis. Analysis of these using TopFIND identified 567 sites that could be attributed to known protease activities, involving meprins, MMPs, chymase, and neutrophil elastase [60]. However, the majority of sites were unattributable to a specific protease, emphasizing the need for acceleration of reverse degradomics.

Human body fluids are well amenable to terminomics analysis despite their high protein dynamic range, which can be mitigated by prefractionation of the sample, depletion of the most abundant proteins such as albumen or fractionation of the peptides after proteome digestion. Sabino et al. identified 9802 neo-termini arising from 2020 proteins in human wound fluid extracted from foam dressings [61]; they reduced proteome complexity by combinatorial peptide ligand libraries (available commercially as Proteominer) [62]. Wildes and Wells used N-terminal labeling by subtiligase to undertake terminomics of human blood, identifying 772 unique N-termini in 222 proteins and sampling across six orders of magnitude of abundance [63].

Das and colleagues performed the first degradomics analysis of synovial fluid and demonstrated that mast cell tryptase degraded a key joint component, proteoglycan-4 or lubricin, which serves as an essential lubricant [64]. Bhutada et al used TAILS to investigate proteolysis in one of the most common diseases associated with tissue breakdown, that is, osteoarthritis (OA). In this condition affecting over 7% of the world’s population, especially adults over 60 years of age, there is extensive bone and cartilage remodeling and progressive loss of articular cartilage. Analysis was first done using OA cartilage [14] and subsequently, matched cartilage and synovial fluid [65] from patients undergoing knee replacement surgery. Proteolytic destruction of osteoarthritic cartilage was found to occur on an unexpectedly massive scale, with nearly 11 000 proteolytic sites identified in cartilage and over 14 000 in synovial fluid, the majority arising in ECM components (in cartilage) and secreted/plasma proteins (in synovial fluid) [14,65]. Analysis of matched cartilage and synovial fluid samples established that proteolytic cartilage breakdown products were transmitted to synovial fluid with 677 peptides from 153 proteins consistently present in both sample types [14,65]. Together with prior work on synovial fluid by Das et al [64], this work supported synovial fluid analysis as a suitable liquid biopsy of the joint for developing clinically useful biomarkers. A striking feature of the OA degradome was pervasive ‘ragging’, that is, stepwise deletion of 1–3 amino acids from peptide termini [14,65]. It accounted for approximately 47% of identified cleavages, suggesting widespread exopeptidase activity alongside endopeptidase cleavage events. Ragging likely reflects the prolonged residence time of proteolytic fragments within the joint, where they may be exposed to exopeptidases over the long OA timeline, which may span several years of gradual cartilage destruction and remodeling.

By integrating forward degradomics of late-stage knee OA cartilage with reverse degradomics of individual proteases HtrA1, ADAMTS5, MMP13, and CMA1, Bhutada and colleagues attributed > 1000 specific cleavages in the OA cartilage degradome to distinct proteolytic enzymes [14,65]. HtrA1, identified as the most abundant protease in these samples, generated 323 cleavages from 109 cartilage proteins [14]. Reverse degradomics of ADAMTS5, a primary aggrecanase, revealed 287 cleavage sites with broad activity against multiple substrates, including COMP, PRELP, and various collagens, although its detection of aggrecan peptides was limited by technical challenges in identifying heavily glycosylated regions [65]. MMP13 cleaved collagen II as well as PRELP, biglycan, and COMP. Notably, the mast cell protease CMA1, whose role in OA was previously undefined, generated 201 distinct cleavages predominantly in COMP, aggrecan, PRELP, and fibronectin [65]. Comparing all four protease degradomes revealed very little overlap in the sites they attacked. Furthermore, even when accounting for ragging-related redundancy, the reverse degradomes of HtrA1, ADAMTS5, MMP13, and CMA1 collectively explained only a fraction of the total cleaved peptides observed in OA cartilage and synovial fluid [65]. This finding strongly suggests that cartilage degradation in human knee OA results from a complex, multiple-protease-mediated degradative process with the fragments accumulating over the long disease timeline. Although ADAMTS5 and CMA1 were not detected in late-stage OA cartilage, the presence of their proteolytic products suggested different protease activities may dominate at distinct phases of disease progression.

HUNTER with a shortened labeling time was used to define the plasma N-terminome in patients with hemolytic uremia syndrome induced by Shiga-toxin E.coli who were undergoing treatment with eculizumab for complement inhibition, comparing plasma samples at baseline and after treatment [66]. This analysis detected 476N-termini from 130 proteins which were differentially abundant, showing that N-terminomics could be effectively applied to study proteolytic impact of drugs using plasma. Next, the plasma N-terminome of patients with systemic lupus erythematosus (SLE) was analyzed using HUNTER optimized with a robot. In most SLE patients, significant changes in proteolytic termini were detected relative to healthy controls, including inter-alpha-trypsin inhibitor heavy chain H1 (ITIH1) a protease inhibitor and PROS1, an immunosuppressive anticoagulant protein [66]. In addition, several N-termini were associated with specific clinical parameters of SLE and the patient trajectory [66]. Reverse degradomics of four proteases active in the human complement system (MASP-1, MASP-3, C1S, C1r) was undertaken by digestion of heat-treated plasma *in vitro*, identifying a number of novel cleavages as well as a significant proportion of cleavages in complement proteins, some of which were detected in SLE plasma [66]. In addition to providing an excellent example of how N-terminomics can be deployed for forward and reverse degradomics, this article made a strong case for expanding degradome research by showing that specific immunoactive fragments that modulate inflammation and tissue injury, such as one from complement C3 characterized therein, can be generated by proteolysis [66].

The limited examples of forward degradomics using human samples will benefit from cross-analysis with reverse degradomics undertaken in human proteome libraries. For example, comparison of cells lacking a protease of interest with the wild-type counterpart has been undertaken for calpain-2 [67], ADAMTS9 [68], and calpain-5 [69]. Anderson et al. defined cleaved sites in human macrophage proteins after chemical inhibition of legumain [70]. TAILS has also been applied for drug testing by reverse degradomics. For example, TAILS of vitreous humor from rabbits and monkeys treated with an HtrA1 inhibitory antibody was used to determine the relevant substrates of HtrA1 and inhibitory efficiency of this antibody [71]. Proteolytic peptides in retinol-binding protein 3 (RBP3), Dickkopf-related protein 3 (DKK3), and clusterin (CLU) were consistently identified in both animal models and were higher in HtrA1-active as compared with HtrA1-inhibited samples.

## Technical limitations and current challenges in DEGRADOMICS

### Caveats and limitations of terminomics

Maximizing proteome yield from tissues for forward degradomics may require harsh conditions, including extremes of pH, which may hydrolyze peptide bonds and addition of chaotropic agents such as guanidine hydrochloride, which denatures proteins and their complexes. Hence, it may be necessary to accept a residual pellet during tissue extraction to ensure minimal protein disruption. Reverse degradomics usually does not employ similar extraction conditions; therefore, proteolytic cleavages in reverse degradomes that match those in forward degradomes may provide confidence for proteolytic origin of the neo-peptide. The dynamic range of ECM (as well as of cells, body fluids, and tissues) means that some exceedingly rare neo-peptides may escape detection despite enrichment.

### Technical limitations

Many technical limitations of degradomics are related to the inherent drawbacks of shotgun proteomics, which is used in most workflows, since proteolysis with trypsin or other proteases is necessary to digest proteins to peptides for detection. As a result, and in contrast to ‘top-down’ proteomics and peptidomics, shotgun experiments typically only identify short peptides with neo-termini, but not entire protein fragments (other than peptides with nontryptic N- and C-termini at each end). Peptides which are larger or smaller than the selected mass window, determined by where trypsin cleavage sites occur in the vicinity of fragment ends, are likely to escape detection. In addition, peptides with complex or unknown modifications (e.g., many forms of glycosylation) typically escape detection unless the modification is specifically allowed for. The large aggregating proteoglycan aggrecan, whose breakdown is important in OA pathogenesis, illustrates some of these challenges. In addition to its 3 globular domains, it has a large central glycosylated region with numerous O- and N-glycans and chondroitin sulfate chains. On shotgun analysis of aggrecan (or the closely related proteoglycan versican), coverage of this region is typically sparse and the known cleavages occurring here were uncovered by Edman degradation. For example, the neo N terminus ARGSVIL results from cleavage at a canonical cleavage site (RNITEGE↓ARGSVIL) by ADAMTS proteases known as aggrecanases. When trypsin is used as the working protease, it will cleave two residues downstream of this cleavage, generating an undetectable dipeptide Ala-Arg, explaining why a neo-N-terminal peptide downstream of the cleavage site has not been detected by TAILS analysis of OA cartilage or cartilage digested with ADAMTS5. Even if this trypsin cleavage does not occur (i.e., a ‘missed’ cleavage), the next Arg/Lys residue is 46 amino acids downstream so that the resulting peptide may be too large to be detected. Upstream of the aggrecanase cleavage site, the peptide with sequence NITEGE is theoretically detectable by LC–MS/MS but was not identified. It is possible that it carries a modification or is further cleaved by another peptidase. In contrast, aggrecan cleavage at the canonical MMP site DIPEN↓FFGVG was detected by identification of a C-terminal peptide with sequence [R].YDAICYTGEDFVDIPEN.[F], whereas the predicted neo-N-terminal peptide, [N].FFGVGGEEDITVQTVTWPDMELPLPR was not detected. These examples illustrate how even with the extensive proteome coverage permitted by LC–MS/MS, the scale of proteolysis in any disease is likely to be severely underestimated.

In top-down proteomics, in contrast, digestion with trypsin or another protease is omitted prior to LC–MS/MS, which means that intact protein isoforms and fragments can be observed. Briefly, the protein ion is first stored for mass measurement and then fragmented to determine its sequence. Top-down proteomics presently has limited relevance for ECM because of the extensive and variable PTMs of ECM components and the formation of complexes by most ECM proteins. It is potentially advantageous for the detection of small proteins which may not generate a sufficient number of appropriate tryptic peptides compared to large proteins.

### Identifying relevant cleavages and measuring dynamics

Since ECM proteins are modular and the modules frequently contain disulfide bonds, not all proteolytic cleavages would lead to fragmentation. An intra-module cleavage may not fragment the protein if the fragments are held together by disulfide bonds (such a cleavage would result in a ‘nick’). Some observed cleavages in reverse degradomics may not be direct substrates of the targeted protease, but indirect, via activation of other proteases, or degradation of inhibitors. For example, it was notable that the well-established site specificity of V8 protease with Glu or Asp at the P1 position was found in only 74% of cleavages observed by V8 digestion of plasma, suggesting that a quarter may have arisen from activation of host proteases [47]. Degradomics does not directly provide information about the kinetics of cleavage, nor can it reliably rank substrate and site preference or how recently cleavage may have occurred, without specific experimental design, such as serial sampling of digests or by use of multiple time points of digestion in reverse degradomics. A time-resolved analysis of the activity of MMP10 reverse degradome was undertaken using multiplexed iTRAQ-TAILS to determine relative proteolytic efficiency and structural accessibility of multiple cleavage events [72]. Stable isotope labeling by amino acids in culture (SILAC) has been used with N-terminomics for a dynamic view of the N-terminome [73], but because labeled amino acids are expensive, it may be more feasible in cell cultures. Tissues from animals fed with specific SILAC labeling regimens could be used for degradomics, but such experiments are unlikely in human disease.

### Indirect proteolysis and ragging

Analysis of a reverse degradome identifies cleavage sites occurring downstream of a protease, but not necessarily its direct substrates owing to the possibility of activation of another protease. Follow-up validation using binary digests of purified protease with purified substrate can confirm that the substrate is cleaved directly rather than indirectly. Ragging is the appearance of stepwise loss of 1–2 amino acids from an N or C terminus, resulting in a ladder that becomes evident when viewing neo-peptides in a forward degradome using annotation by sequence number. It is rarely seen in reverse degradomes with endopeptidases, suggesting a role for exopeptidases in further modification of endopeptidase cleavages in tissue/disease proteomes. Activity of endopeptidases acting in close proximity can potentially contribute to the complexity of distinguishing primary cleavages from ragging. One of the benefits of undertaking reverse degradomics is that it identifies the primary endopeptidase cleavages from which ragging may be initiated.

## Translation of DEGRADOMES for human disease and future developments

Beyond expanding knowledge of human disease, tissue turnover and protease mechanisms, human degradomics has practical value for diagnosis and treatment. Several ECM-derived matrikines, which are proteolytic fragments with bioactivity are known, for example, endostatin from collagen XVIII [74], endotrophin from the a3 chain of collagen VI [75], and versikine from versican [76]. Peptides with known neo-termini have provided immunogens that can be used to generate antibodies highly specific for cleaved termini but not intact sequences. Such neoepitope antibodies have been generated against a number of termini, proving their utility beyond a doubt, and are versatile reagents that can be used in immunostaining, western blotting and elisa. Examples include neoepitope antibodies to cleavages in the proteoglycans aggrecan [44,77], versican [78] and brevican [79], collagens [80,81] and cartilage oligomeric matrix protein (COMP, or thrombospondin 4) [82].

Systematic analysis of human diseases is likely to uncover associations of neo-termini with distinct patterns of proteolysis in human diseases. Clusters of disease-associated neo-termini could be used to generate targeted proteomics assays referred to as multiple reaction monitoring (MRM) or parallel reaction monitoring (PRM). Proteases strongly associated with disease can be targeted by drugs, providing new therapeutic approaches. For now, analyses of cell cultures and mouse models of diseases outnumber those using human tissues and body fluids. However, the necessary elements are in place for systematically mapping the degradomes of different diseases, that is, diverse N-terminomics and C-terminomics workflows, high-resolution mass spectrometers with high-throughput, powerful data analytic packages and routine submission of datasets to ProteomeXchange as a condition of publication.

This ecosystem makes possible consideration of a *human degradome project*, a purpose-driven initiative to map the proteolytic landscapes of a variety of human diseases. It has the potential to provide an abundance of new biomarkers for precision medicine, mechanistic knowledge of protease activities (via reverse degradomics) and identify proteases as relevant drug targets. It would allow determination of overlap and contrasts in disease degradomes, help stratify disease subtypes and elucidate chronic disease timelines, define protease activities across diseases, test inhibitors and help predict inhibitor effects—especially potential side effects at nondisease sites. A knowledgebase on this scale would be transformative for the global medical research community and is a desirable objective for acceleration of diagnostics and medicines. Such an initiative could be implemented for proof of concept and feasibility on a small scale in a single laboratory using a limited variety of diseased tissues or body fluids with public access to data but better implemented by collaborating groups worldwide. It would require consideration and prioritization of the most common disease states (e.g., inflammatory conditions, cancers, degenerative disorders), inclusion of diverse tissue origins (by organ, e.g., breast cancer, colon cancer) and selection of appropriate representative patients by expert clinicians. On the technical side, the project would be facilitated by automated sample handling, access to high-resolution mass spectrometers, a variety of LC–MS/MS data analysis tools and computer hardware and software for storage, retrieval and online access. All these resources exist, but successful implementation will require bringing them together, ideally by collaboration of the interested parties and dedicated funding.

## Figures and Tables

**Table 1.** Degradomics workflows.

Workflow name	N-term/C-term	Enrichment	Label	Fractionation	Proteome digestion

TAILS [2]	N-term	Negative (HPG-ALD)	TMT, dimethyl		Trypsin
COFRADIC [83]	N-term	Negative		SCX	Trypsin
ChaFRADIC [84]	N-term	Negative		SCX	Trypsin
HUNTER [85]	N-term	Negative (Undecanal)	TMT, dimethyl		Trypsin
HYTANE [86]	N-term	Negative (hexadecanal)	Dimethyl, TMT		Trypsin
P-TAG [87]	N-term	Negative (TiO2 affinity)	TMT, dimethyl, GAP3		Trypsin
TAGS-CR [88]	N-term	Negative		SCX	Trypsin
CHAMP-N [89]	N-term	Negative		SCX	LysargiNase (TrypN)
FAIMS [58]	N-term	None	TMT, dimethyl	FAIMS	Trypsin
Biotinylation-subtiligase [63]	N-term	Positive (biotin-avidin)	Subtiligase		Trypsin
N-CLAP [90]	N-term	Positive(biotin-avidin)	PITC in TFA		Trypsin
CHOPS [91]	N-term	Positive (Streptavidin)	2PCA		Trypsin
Immuno-N-terminomics (miNterm)	N-term	Positive (TMT resin)	TMT		Trypsin
PICS2 [92]	N-term	Positive (neutravidin)	disulfide-2PCA		Trypsin
CHOPPER	N-term	Positive (neutravidin)	alkene-2PCA		Trypsin
C-COFRADIC [93]	C-term	Negative (amine-reactive butyrate)		SCX	Trypsin
CPB-ChaFRADIC [94]	C-term			SCX	Carboxypeptidase B
C-TAILS [95,96]	C-term	Negative (amine polymer)	EDC		Trypsin
LysC digestion, dimethylation and enrichment [97]	C-term	Negative (NHS resin)	2PCA		LysC
PBC	C-term	Negative (Streptavidin)	2PCA		Trypsin
MOLEX chromatography [98]	C-term	Negative		MOLEX	GluC (V8)

**Table 2.** Resources for degradome and proteome data analysis.

Package	Function

Proteome discoverer	DDA and DIA data analysis for Thermo Fisher Mass spectrometers
Spectronaut [99]	DDA and DIA data analysis and can analyze data from various mass spectrometers
MaxQuant [100]	DDA and DIA data analysis and can analyze data from various mass spectrometers
Trans Proteomic Pipeline [29]	DDA and DIA data analysis and can analyze data from various mass spectrometers
FragPipe [21]	DDA and DIA data analysis and can analyze data from various mass spectrometers
DIA-NN [22]	DIA data analysis and can analyze data from various mass spectrometers
Skyline [101]	DDA and DIA data analysis and can analyze data from various mass spectrometers
OpenSwath [102]	DIA data analysis and can analyze data from various mass spectrometers
UniProtKB	Provides reference protein database validated protein sequences and structure, domains. PTMs, cellar compartment annotations, mapped alternate cleavage sites, natural processing sites, and isoform information.
MANTI [26]	Peptide annotation for terminomics using MaxQuant output.
TermineR [27]	Peptide annotation for terminomics using FragPipe output
CLIPPER [28,30]	Peptide annotation for terminomics using Proteome Discoverer and Spectronaut. It can also be manually customized for other softwares. Create visualizations and perform statistics.
Perseus [31]	Create visualizations and perform statistics. Requires manually curated dataset for terminomics.
Proteasy [103]	R package to retrieve data from MEROPS for substrates and proteases of interest.
MEROPS [33]	Protease and protease inhibitors database. Experimentally validated substrate and cleavage site information.
TopFind [34]	Integrated data from UniProt, MEROPS and community submissions from experimental terminomics datasets. Includes PathFINDer for pathway analysis predictions.
DICED [35]	Experimentally generated forward and reverse N-terminomics datasets for disease-specific protease activity patterns.
CapSites [104]	Database for experimentally generated human caspase cleavage sites.

## Data Availability

This is a literature review and opinion article. Therefore, no data was generated or presented.

## Abbreviations

2PCA: 2-pyridinecarboxaldehyde
ADAM: A disintegrin and metalloprotease domain
ADAMTS: A disintegrin-like and metalloprotease domain with thrombospondin type 1 motif
CHA-FRADIC: charge-based fractional diagonal chromatography
CHAMP-N: chromatographic amplification of protein N-terminal peptides
CHOPPER: chemical enrichment of protease substrates with purchasable elutable reagents
CHOPS: chemical enrichment of protease substrates
COFRADIC: combined fractional diagonal chromatography
CPB-ChaFRADIC: Carboxypeptidase B-assisted charge-based fractional diagonal chromatography
DDA: data-dependent acquisition
DIA: data-independent acquisition
DIA-NN: data-independent acquisition neural-network
DICED: database of identified cleavage sites endemic to diseases
ECM: extracellular matrix
EDC-: 1-ethyl-3-(3-dimethylaminopropyl) carbodiimide
FAIMS: field asymmetric ion mobility spectrometry
GAP3: glyceraldehyde-3-phosphate
HPG-ALD: hyperbranched polyglycerol aldehydes
HUNTER: high-efficiency undecanal-based N-termini enrichment
HYTANE: hydrophobic tagging-assisted N-termini enrichment
iTRAQ: isobaric tag for relative and absolute quantitation
LC–MS/MS: liquid chromatography tandem mass spectrometry
MANTI: Maxquant advanced N-termini interpreter
MMP: matrix metalloproteinase
MOLEX: molecular extraction
MRM: multiple reaction monitoring
N-CLAP: N-terminalomics by chemical labeling of the alpha-amine of proteins
NHS: N-hydroxy succinimide
OA: osteoarthritis
PBC: 2-PCA- and biotin labeling–based C-terminomics
PICS-2: proteomic identification of cleavage sites with 2PCA reagents
PITC: phenyl isothiocyanate
PRM: parallel reaction monitoring
P-TAG: phosphor-tagging
PTM: post-translation modification
RT-qPCR: reverse transcriptase-quantitative polymerase chain reaction
SARS-CoV2: severe acute respiratory syndrome coronavirus 2
SCX: strong cation exchange
SILAC: stable isotope labeling by amino acids in culture
SLE: systemic lupus erythematosus
TAGS-CR: terminal amine guanidination of substrates-charge reversal
TAILS: terminal amine isotopic labeling of substrates
TFA: trifluoracetic acid
TMT: tandem mass-tags
